# Impact of a multimodal antimicrobial stewardship intervention on fluoroquinolone usage for antimicrobial prophylaxis before urologic procedures in a Veterans Affairs outpatient clinic

**DOI:** 10.1017/ash.2025.10175

**Published:** 2025-10-14

**Authors:** Gertrude Kinyua, Gaielle Harb, Teri Hopkins, Christopher Frei, Vidal Mendoza, Jose Cadena-Zuluaga, Elizabeth Walter

**Affiliations:** 1 South Texas Veterans Health Care System, Department of Pharmacy, San Antonio, TX, USA; 2 University of Texas at Austin, College of Pharmacy, Austin, TX, USA; 3 University of Texas Health Science Center at San Antonio, Joe R. and Teresa Lozano Long School of Medicine. San Antonio, TX, USA

## Abstract

**Objective:**

This study evaluated the impact of a multimodal antimicrobial stewardship intervention on fluoroquinolone (FQ) use for prophylaxis in outpatient urologic procedures.

**Methods:**

This single-center retrospective cohort study included patients from the South Texas Veterans Affairs (VA) outpatient urology clinic who underwent procedures between December 1, 2020, and February 29, 2024. Interventions included academic detailing, provider-specific FQ use reports, and prospective urine culture reviews with feedback. One pre-intervention cohort (PRE) and three post-intervention cohorts (POST2021, POST2022, POST2023) were analyzed. The primary outcome was FQ days of therapy (DOT); secondary outcomes included inappropriate prescriptions, post-operative complications, emergence of FQ resistance within 1 year, and *Clostridioides difficile* infection within 30 days of prophylaxis.

**Results:**

This analysis included data from 548 patients (150 PRE, 139 POST2021, 168 POST2022, 91 POST2023). Median age was similar across groups (*p* = 0.20), with over 90% male in each cohort (*p* = 0.07). Over one-third in each cohort received pre-operative oral antibiotics, 25% of which were FQs. More than 90% received pre-operative IV antibiotics, and over 50% received post-operative oral antibiotics. A significant reduction in FQ DOT/100 procedures was noted from pre- to post-intervention groups (98.6 PRE, 49.6 POST2021, 53.5 POST2022, 45.1 POST2023). No significant differences were observed in the secondary clinical outcomes.

**Conclusion:**

A multimodal stewardship initiative reduced FQ use before urologic procedures, mainly due to decreased IV use. Further efforts are needed to optimize pre-operative FQ use and address drivers of post-operative antibiotic prescribing.

## Introduction

Antimicrobial prophylaxis prior to urologic procedures is routinely used to minimize the risk of surgical site infections (SSIs) and postoperative urinary tract infections (UTIs), which are common causes of patient morbidity.^1^ In the 2008 American Urological Association (AUA) guidelines, fluoroquinolones (FQs) were strongly recommended for prophylaxis in this setting; however, the updated 2019 guidelines recommend a broader range of agents, including beta-lactams and sulfonamides, with FQs now primarily reserved as an option for transrectal prostate biopsy.^1,2^ FQs were frequently prescribed in this setting due to their antimicrobial activity against local organisms in the genitourinary system, attainment of tissue concentration higher than most MIC_90_ values for the common uropathogens, and long half-life that maintains sufficient serum and tissue concentrations for the duration of the procedure without the need for redosing.^3^ However, from 2008 to 2018, the FDA added boxed warnings for serious adverse effects, and their use is also linked to development of extended-spectrum beta-lactamases (ESBLs), and *Clostridioides difficile*-associated diarrhea (CDAD).^4–8^

Using the shortest effective duration of antimicrobial prophylaxis is important to prevent adverse events associated with antimicrobials and reduce emergence of multidrug-resistant pathogens, since systemic antimicrobial usage is the primary driver of antimicrobial resistance.^1^ Both the AUA and the Infectious Diseases Society of America (IDSA) guidelines recommend a short duration of antimicrobial prophylaxis (1–2 doses) before urological procedures, regardless of presence of asymptomatic bacteriuria (ASB).^1,9^ Additionally, published literature suggests that a short duration of antimicrobial prophylaxis is safe and effective before urological procedures.^10–12^ A prospective, randomized study found single-dose prophylaxis to be as effective as treatment until urine sterilization in patients with ASB undergoing urologic surgical procedures.^10^

At the South Texas Veterans Healthcare System (STVHCS), an analysis of FQ usage in the outpatient setting between December 2020 and May 2021 found that urology accounted for 42% of institutional FQ usage. The majority of these prescriptions were for ≥7 duration of therapy for ASB prior to a urological procedure. In response, the antimicrobial stewardship program (ASP) implemented a multimodal, non‑restrictive initiative aimed at reducing FQ prophylaxis in urologic procedures. Nonrestrictive strategies targeting inappropriate FQ use have been described in literature with favorable outcomes.^13,14^ Specific nonrestrictive interventions implemented at our institution included provider education, individualized feedback on antimicrobial prescribing, and prospective audit and feedback of urine cultures. This study evaluated the effectiveness of these interventions in decreasing FQ use at our institution.

## Methods

### Antimicrobial stewardship actions that enabled this analysis

In May 2021, academic detailing was conducted by an infectious diseases (ID) pharmacist that included education on changes to local UTI treatment guidelines. In July 2022, data on FQ usage, including individual provider usage and duration of therapy by urology provider, was also collected and sent to the chief of urology. The intent was to facilitate targeted discussions with providers demonstrating high FQ usage. Starting in April 2023, an ID pharmacist performed prospective reviews of preoperative urine cultures, followed by feedback on optimal pathogen-directed therapy, including education regarding the risks, benefits, and appropriate use of FQ and appropriate duration of therapy. Individual urology providers were contacted for academic detailing in July of 2023, followed by finalized large-group academic detailing in August of 2023 that included a review of urology prescribing data for preprocedural antibiotics, recommendations for the use of antimicrobials for ASB in patients undergoing urologic procedures, and proposed strategies to reduce FQ utilization.

### Study design

This was a single-center, retrospective, cohort study of patients receiving antimicrobial prophylaxis for urologic procedures at STVHCS outpatient urology clinic. The study was reviewed by the University of Texas Health Science Center at San Antonio Institutional Review Board (San Antonio, TX, USA), through the Office of Clinical Research, and was approved under non-regulated research. It was a retrospective chart review investigation of interventions that had already been made under our local antimicrobial stewardship program as quality improvement; as such, informed consent was waived. All methods were performed in accordance with the *institution relevant guidelines and regulations.*

### Data collection

**Figure 1.**
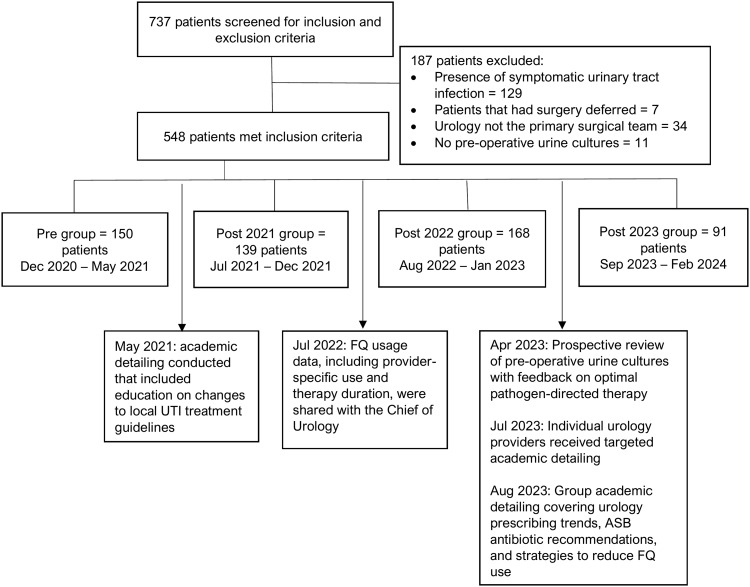
Outline of study cohorts’ development and timeline of antimicrobial stewardship interventions.

Patients were identified through the Structured Query Language database by ICD-10-CM diagnostic codes related to urology procedures. A retrospective chart review was performed on all outpatient visits during 4 time periods: one pre-intervention (PRE: 12/2020–05/2021) and 3 post-intervention: (POST2021: 07/2021–12/2021), (POST2022: 08/2022–01/2023), and (POST2023: 09/2023–02/2024). Time periods correspond to a six-month interval before intervention implementation for the PRE group and six-month intervals after implementation of an intervention for the POST groups (Figure 1). Data on patient demographics, underlying comorbidities, microbiological results, antimicrobial regimens, and outcomes were collected manually from the computerized patient record system (CPRS) electronic medical record.

### Study population

Patients were included if they were 18 years of age or older and underwent a urologic procedure in the outpatient setting. Patients were excluded for one or more of the following criteria: (1) presence of symptomatic UTI, (2) delayed/deferred surgery (3) urology was not the primary surgical team, or (4) no preoperative urine cultures were obtained/recorded.

### Outcomes

The primary outcome was FQ days of therapy (DOT), normalized per 100 urologic procedures. Secondary outcomes included percentage of inappropriate prescriptions, duration of therapy of preoperative prescriptions, rates of 30-day postoperative UTIs, SSI, and bloodstream infections (BSI), emergence of FQ resistance within 1 year after the procedure, and *Clostridioides difficile* infection (CDI) within 30 days after completion of antimicrobial prophylaxis.

### Definitions

Symptomatic UTIs were defined as at least 1 clinical criterion or ≥2 laboratory/microbiological criteria without any clinical criteria. Acute pyelonephritis was defined as acute flank pain or costovertebral angle tenderness and at least 1 clinical criterion of UTI reported. Clinical criteria included dysuria, hematuria, increased frequency, suprapubic or pelvic pain, chills, fever, flank pain, and altered mental status. Laboratory/microbiological criteria included pyuria (>10 WBC/mm^3^), leukocytosis (WBC > 10,000/mm^3^), and positive urine culture (10^5^ CFU/mL or greater). Inappropriate prescriptions were defined as antibiotics prescribed with cultures indicating organisms resistant to the prescribed antibiotic or prescriptions prescribed for >2-day duration of therapy in the absence of indication necessitating prolonged duration such as UTI or pyelonephritis. Emergence of FQ resistance was defined as isolation of a FQ-resistant organism from any site that was not present before receipt of FQ for prophylaxis. Procedures were classified as low-, intermediate-, high-risk, or as yet undetermined probability for an associated SSI per the AUA proposed procedure-associated risk probability of SSI.^1^

### Statistical analysis

DOT was based on patient-level exposure and was defined as the aggregate sum of days that a patient was on a fluoroquinolone, regardless of dose or frequency. While outcomes were reported individually for each cohort, statistical comparisons were performed between the preintervention cohort and all the postintervention cohorts. Descriptive statistics were used to summarize data. Analysis of categorical data was conducted using χ^2^ or Fisher’s Exact test. Wilcoxon rank sum and Kruskal–Wallis tests were used to analyze continuous data, since the continuous data were not normally distributed. To detect a difference of 1.0 day in FQ DOT, with an 80% power and a two-sided alpha of 0.05, 65 patients were required for each group. JMP 17.0 statistical software (SAS Institute, Cary, NC) was used for all analyses.

## Results

Of 737 screened patients, 548 met study criteria: *n* = 150 (PRE), *n* = 139 (POST2021), *n* = 168 (POST2022), and *n* = 91 (POST2023). Notably, the POST2023 cohort was considerably smaller than other cohorts, primarily due to limitations on the number of elective procedures during this period, resulting from shortages in surgical supplies and staffing. The median age was similar across groups (*P* = 0.20), and over 90% of patients in each group were male (91% PRE, 97% POST2021, 96% POST2022, 96% POST2023; *P* = 0.07). The majority of procedures were classified as intermediate-risk (66% PRE, 66% POST2021, 66% POST2022, 73% POST2023; *P* = 0.34) per the AUA proposed procedure-associated risk probability of SSI. Overall, 29 patients underwent transrectal prostate biopsies (3% PRE, 4% POST2021, 8% POST2022, 8% POST2023) (Table 1). Approximately one-third of patients across all cohorts had positive preoperative urine cultures. Of these, 35% in the entire cohort were polymicrobial (40% PRE, 48% POST2021, 25% POST2022, 26% POST2023; *P* = 0.15). Table 1 contains a complete description of baseline characteristics, urologic conditions, and classification of procedure-associated risk probability of surgical site infection. Table 2 contains urine culture results.

**Table 1.** Baseline characteristics, urologic conditions, classification of procedure-associated risk, probability of surgical site infection, and transrectal prostate biopsy

Characteristics	Entire cohort (*n* = 548)	PRE (*n* = 150)	POST21 (*n* = 139)	POST22 (*n* = 168)	POST23 (*n* = 91)	*P* value
Age, years, median (IQR)	67 (59–74)	69 (62–74)	66 (58–73)	67 (59–74)	68 (58–75)	.20
Male, n(%)	519 (95)	136 (91)	135 (97)	161 (96)	87 (96)	.07
BMI, kg/m^2^, median (IQR)	30 (27–34)	30 (27–33)	30 (27–34)	30 (26–34)	31 (26–34)	.95
Diabetes, n (%)	217 (40)	54 (36)	50 (36)	70 (42)	43 (53)	.25
Renal insufficiency, n (%)	89 (16)	31 (21)	16 (12)	28 (17)	14 (15)	.21
**Urologic conditions**						
BPH, n(%)	105 (19)	22 (15)	31 (22)	25 (15)	27 (30)	.01^(a)^
Renal/ureteral stent, n(%)	9 (2)	0 (0)	9 (6)	0	0	--^*^
Renal stone, n(%)	80 (15)	18 (12)	36 (26)	13 (8)	13 (14)	<.01^(b)^
Prostate cancer, n(%)	105 (19)	21 (14)	44 (32)	19 (11)	21 (23)	<.01^(c)^
Bladder/ureteral cancer, n(%)	58 (11)	19 (13)	21 (15)	10 (6)	8 (9)	.05^(d)^
Urethral stenosis/stricture, n(%)	32 (6)	8 (5)	14 (10)	5 (3)	5 (5)	.07
Indwelling catheter, n(%)	12 (2)	0	9 (6)	0	3 (3)	--^*^
Recurrent UTI, n(%)	17 (3)	0	9 (6)	0	8 (9)	--^*^
Other, n(%)	273 (50)	69 (46)	59 (42)	101 (60)	44 (8)	.01^(e)^
**AUA Proposed procedure-associated risk probability of surgical site infection**						
Low risk, n(%)	44 (8)	11 (7)	15 (11)	10 (6)	8 (9)	.46
Intermediate risk, n(%)	360 (66)	99 (66)	92 (66)	103 (61)	66 (73)	.34
High risk, n(%)	91 (17)	31 (21)	14 (10)	35 21)	11 (12)	.02^(f)^
As yet undetermined, n(%)	53 (10)	9 (6)	18 (13)	20 (12)	6 (7)	.11
**Transrectal Prostate Biopsy**						
Transrectal prostate biopsy, n(%)	29 (5)	4 (3)	5 (4)	13 (8)	7 (8)	--^*^

* *P* value not reported as 20% of cells have expected count less than 5 hence χ^2^ test may not be valid.

Abbreviations: BPH, benign prostatic hyperplasia; UTI, urinary tract infection.

Other category included conditions such infertility, incontinence, erectile dysfunction, elevated prostate-specific antigen and Peyronie’s disease, among others.

(a) statistically significant difference observed between PRE and POST2023 group (*P* < .01).

(b) statistically significant difference observed between PRE and POST2021 group (*P* < .01).

(c) statistically significant difference observed between PRE and POST2021 group (*P* < .01).

(d) statistically significant difference observed between PRE and POST2022 group (*P* = .03).

(e) statistically significant difference observed between PRE and POST2022 group (*P* = .02).

(f) statistically significant difference observed between PRE and POST2021 group (*P* = .01).

**Table 2.** Urine cultures

	Entire cohort (*n* = 548)	PRE (*n* = 150)	POST21 (*n* = 139)	POST22 (*n* = 168)	POST23 (*n* = 91)	*P* value
Culture negative, n (%)	365 (67)	100 (67)	93 (67)	112 (67)	60 (66)	.38
Culture positive, n (%)	183 (33)	50 (33)	46 (33)	56 (33)	31 (34)	.99
Bacterial, n (%)	180 (98)	49 (98)	46 (100)	54 (96)	31 (100)	.99
Gram positive, n (%)	107 (59)	36 (73)	23 (50)	27 (50)	21 (23)	.19
Gram negative, n (%)	119 (66)	27 (55)	35 (76)	38 (70)	19 (21)	.51
Polymicrobial^(a)^, n (%)	64 (35)	20 (40)	22 (48)	14 (25)	8 (26)	.15
Fungal, n (%)	5 (3)	1 (2)	0	3 (5)	1 (3)	.42

(a) polymicrobial was defined as isolation of more than one microorganism from urine cultures.

A total of 194 (35%) patients in the entire cohort received preoperative oral antimicrobials (Table 3). FQs accounted for one-quarter of these prescriptions, and rates were identical across the cohorts. The most frequently prescribed days’ supply was a 7-day supply (70% PRE, 78% POST2021, 80% POST2022, 77% POST2023; *P* = 0.79). In the entire cohort, 97% of patients also received preoperative intravenous (IV) antimicrobials (Table 3). FQs accounted for 48% of these prescriptions in the pregroup, with a significant decrease observed in the post groups (48% PRE, 14% POST2021, 12% POST2022, 16% POST2023; *P* < 0.01). On the other hand, a statistically significant increase in preoperative IV cephalosporin use was observed across the cohorts. Overall, 31% of patients in the entire cohort received both oral and IV preoperative antibiotics. In addition to preoperative antimicrobials, the majority of patients also received postoperative antimicrobial prophylaxis (62% PRE, 57% POST2021, 70% POST2022, 54% POST2023; *P* = 0.21). Notably, there was a statically significant decrease in postoperative FQ usage observed (34% PRE, 19% POST2021, 17% POST2022, 1% POST2023; *P* < 0.01) (Table 4).

**Table 3.** Preoperative antimicrobials

	Entire cohort (*n* = 548)	PRE (*n* = 150)	POST21 (*n* = 139)	POST22 (*n* = 168)	POST23 (*n* = 91)	*P* value
**Preoperative oral antimicrobials**						
Preoperative oral antibiotics, n (%)	194 (35)	54 (36)	45 (32)	66 (39)	35 (38)	.73
Fluoroquinolone	45 (23)	13 (24)	10 (22)	14 (21)	8 (23)	.96
Penicillin	63 (39)	10 (24)	13 (37)	23 (44)	17 (59)	.03
Cephalosporin	20 (13)	4 (8)	9 (26)	6 (12)	1(3)	.06
Sulfonamide	33 (20)	8 (20)	7 (20)	10 (19)	8 (28)	.79
Nitrofuran	46 (29)	16 (39)	12 (34)	11 (21)	7 (24)	.22
Other	14 (9)	4 (8)	3 (9)	6 (12)	1 (3)	.68
Days’ supply, n (%)						
1–2 days	19 (10)	4 (7)	5 (11)	6 (9)	4 (11)	.92
3–5 days	14 (7)	5 (9)	5 (11)	1 (2)	3 (9)	.21
≥ 7 days	155 (80)	38 (70)	35 (78)	53 (80)	27 (77)	.79
≥ 14 days	6 (3)	2 (4)	1 (2)	2 (3)	1 (3)	.96
**Preoperative intravenous antimicrobials**						
Preoperative intravenous antibiotics, n (%)	533 (97)	148 (99)	136 (98)	157 (93)	91 (100)	<.01
Fluoroquinolone	125 (23)	72 (48)	19 (14)	19 (12)	15 (16)	<.01
Penicillin	14 (3)	3 (2)	5 (4)	5 (3)	1 (1)	--^*^
Cephalosporin	256 (48)	36 (24)	84 (62)	92 (59)	44 (48)	<.01
Aminoglycoside	133 (25)	37 (25)	31 (23)	32 (20)	33 (36)	.04
Vancomycin	140 (26)	39 (26)	28 (21)	41 (25)	33 (36)	.07
Clindamycin	7 (2)	1 (.7)	2 (1.4)	4 (2.5)	0	.31
Azole	1 (.2)	0	0	1 (.6)	0	--^*^
Both oral and IV preoperative antibiotics, n (%)	171 (31)	43 (29)	47 (34)	50 (30)	31 (34)	.71

* *P* value not reported as 20% of cells have expected count less than 5 hence χ^2^ test may not be valid.

**Table 4.** Postoperative oral antimicrobial

	Entire cohort (*n* = 548)	PRE (*n* = 150)	POST21 (*n* = 139)	POST22 (*n* = 168)	POST23 (*n* = 91)	*P* value
Postoperative antibiotics, n (%)	349 (64)	93 (62)	79 (57)	117 (70)	58 (64)	.21
Fluoroquinolone	68 (19)	32 (34)	15 (19)	20 (17)	1 (1)	<.01
Penicillin	27 (8)	7 (8)	3 (4)	11 (9)	6 (10)	.41
Cephalosporin	86 (25)	10 (11)	19 (24)	43 (37)	14 (24)	<.01
Sulfonamide	140 (40)	40 (43)	28 (35)	38 (32)	34 (59)	.01
Nitrofuran	40 (12)	3 (3)	20 (25)	14 (12)	3 (5)	<.01
Other	13 (3)	5 (5)	0 (0)	8 (7)	1 (1)	.07

Although not statistically significant, preoperative FQ use decreased from 98.6 DOT/100 procedures (PRE) to 49.6 DOT/100 procedures (POST2021), 53.5 DOT/100 procedures (POST2022), and 45.1 DOT/100 procedures (POST2023) (Table 5). This decrease was primarily driven by preoperative IV FQ usage, as minimal impact was observed in preoperative oral FQ usage. Despite the antimicrobial stewardship interventions, inappropriate antimicrobial prophylaxis use remained high (92% PRE, 89% POST2021, 90% POST2022, 89% POST2023; *P* = 0.92) and was entirely driven by inappropriate duration of therapy. No clinically significant differences were observed in any of the secondary outcomes assessed (Table 5).

**Table 5.** Primary outcomes: FQ DOT/100 procedures and secondary outcomes: therapy duration, postoperative infectious complications, and FQ resistance

	PRE (*n* = 150)	POST21 (*n* = 139)	POST22 (*n* = 168)	POST23 (*n* = 91)	*P* value
**Primary outcome**					
FQ DOT/100 procedures	98.6	49.6	53.5	45.1	.27
**Secondary outcomes**					
≥3-day duration of therapy^(a)^, n (%)	45 (92)	40 (89)	56 (90)	31 (89)	.92
1 to -2-day duration of therapy^(a)^, n (%)	4 (8)	5 (11)	6 (10)	4 (11)	.92
Inappropriate prescriptions, n (%)	45 (92)	40 (89)	56 (90)	31 (89)	.92
Inappropriate duration of therapy	45 (92)	40 (89)	56 (90)	31 (89)	--
Inappropriate drug selection due to resistance	0	0	0	0	--
UTI postop, n (%)	18 (12)	20 (14)	16 (10)	8 (9)	.48
Surgical site infection, n (%)	2 (1.3)	0	8 (5)	0	--^*^
BSI, n (%)	1 (.7)	2 (1.4)	0	0	--^*^
Emergence of FQ resistance, n (%)	3 (2)	0	0	0	--^*^
CDAD, n (%)	0	0	0	0	--

* *P* value not reported as 20% of cells have expected count less than 5 hence χ^2^ test may not be valid.

(a) Duration of therapy was defined as the total number of days of intravenous and oral preprocedure antibiotics prescribed.

Abbreviations: DOT, days of therapy; UTI, urinary tract infection; BSI, blood stream infection; FQ, fluoroquinolones; CDAD, *Clostridioides difficile*-associated diarrhea.

## Discussion

This study evaluated the effectiveness of several ASP interventions in a large cohort of patients in a setting where prescribing practices are not well defined. Our results demonstrate that ASP efforts at our institution were associated with reduced IV FQ utilization prior to urologic procedures. The multimodal approach implemented was comprised of provider education, individualized feedback on antimicrobial prescribing, and prospective audit and feedback of urine cultures as core interventions. Although such strategies have been described, the published literature on nonrestrictive stewardship approaches remains limited in comparison to that on restrictive approaches. Troung et al. conducted a retrospective study at two nonacademic community hospitals examining nonrestrictive antimicrobial stewardship interventions.^13^ They found that this approach significantly reduced levofloxacin use, increased ceftriaxone use, and improved *Pseudomonas aeruginosa* susceptibility to levofloxacin. Although restrictive strategies have shown effectiveness in reducing FQ utilization, resistance rates, and *C. difficile* infections,^15–18^ there is often a negative connotation perceived with this approach. For this reason, our ASP program chose nonrestrictive alternatives to mitigate potential negative perceptions among prescribers.

Despite multiple ASP interventions, there was minimal impact on percentage of inappropriate prescriptions, which was entirely driven by inappropriate duration of therapy. Providers were generally receptive to recommendations regarding antibiotic selection; however, recommendations on duration were less frequently followed. Notably, acceptance or rejection of these recommendations was not systematically documented. Furthermore, as IV FQ utilization decreased, a corresponding increase in IV cephalosporin was observed, while oral FQ use remain largely unchanged. The stewardship efforts implemented focused on reducing FQ use, which may have influenced a shift in prescribing practices away from IV FQs prior to procedures. Antimicrobial selection and duration recommendations were primarily communicated to mid-level practitioners and urology fellows, who often expressed that the attendings were driving these decisions. This likely limited the impact of the intervention and may explain the modest effect observed on oral FQ usage. Notably, despite the increase in IV cephalosporin use, there was no observed increase in rates of postoperative infectious complications. Several studies have now assessed the efficacy of non-FQ-based antibiotic prophylaxis and provided evidence to support their use.^19–21^

The high rate and long duration of postoperative antimicrobial prophylaxis were unexpected study findings, as post operative antimicrobials are not recommended in the AUA guidelines.^1^ Consistent with preoperative trends, we observed a statistically significant decrease in postoperative FQ use and a corresponding significant increase in cephalosporin use. Our interventions were not originally intended to target postoperative prophylaxis, and further analysis are needed to identify factors driving postoperative antimicrobial use.

This study has potential limitations. First, the study included mostly male veterans from a single institution; therefore, the results might not be generalizable to all settings. Second, the retrospective and observational nature of this study limits our ability to identify which patient factors might have impacted antimicrobial selection across the cohorts. Third, we did not assess the emergence of resistance to other antibiotic classes, which might have been impacted by increased use of non-FQ-based antibiotic prophylaxis. Fourth, several statistically significant differences were observed in baseline urologic conditions and in the classification of procedure-associated risk for surgical site infection, potentially introducing confounding bias. Given the retrospective design of the study, the underlying reasons for these differences could not be determined, and unmeasured confounding may have influenced the results.

## Conclusion

In this single-center, retrospective cohort study of patients presenting to a VA outpatient urology clinic, a multimodal stewardship initiative was associated with less IV FQ usage prior to urologic procedures. To sustain these efforts, order sets targeting prophylaxis prior to urologic procedures have been implemented to promote guideline-based prescribing and minimize unnecessarily prolonged antibiotic courses. Additional efforts are warranted to improve rates of appropriate preoperative FQ use and identify and mitigate factors driving postoperative antimicrobial use.
